# Medical Image-Based Computational Fluid Dynamics and Fluid-Structure Interaction Analysis in Vascular Diseases

**DOI:** 10.3389/fbioe.2022.855791

**Published:** 2022-04-27

**Authors:** Yong He, Hannah Northrup, Ha Le, Alfred K. Cheung, Scott A. Berceli, Yan Tin Shiu

**Affiliations:** ^1^ Division of Vascular Surgery and Endovascular Therapy, University of Florida, Gainesville, FL, United States; ^2^ Department of Biomedical Engineering, University of Utah, Salt Lake City, UT, United States; ^3^ Division of Nephrology and Hypertension, Department of Internal Medicine, University of Utah, Salt Lake City, UT, United States; ^4^ Veterans Affairs Salt Lake City Healthcare System, Salt Lake City, UT, United States; ^5^ Vascular Surgery Section, Malcom Randall Veterans Affairs Medical Center, Gainesville, FL, United States

**Keywords:** computational fluid dynamics (CFD), finite element analysis, fluid-structure interaction (FSI), patient-specific analysis, image-based simulation

## Abstract

Hemodynamic factors, induced by pulsatile blood flow, play a crucial role in vascular health and diseases, such as the initiation and progression of atherosclerosis. Computational fluid dynamics, finite element analysis, and fluid-structure interaction simulations have been widely used to quantify detailed hemodynamic forces based on vascular images commonly obtained from computed tomography angiography, magnetic resonance imaging, ultrasound, and optical coherence tomography. In this review, we focus on methods for obtaining accurate hemodynamic factors that regulate the structure and function of vascular endothelial and smooth muscle cells. We describe the multiple steps and recent advances in a typical patient-specific simulation pipeline, including medical imaging, image processing, spatial discretization to generate computational mesh, setting up boundary conditions and solver parameters, visualization and extraction of hemodynamic factors, and statistical analysis. These steps have not been standardized and thus have unavoidable uncertainties that should be thoroughly evaluated. We also discuss the recent development of combining patient-specific models with machine-learning methods to obtain hemodynamic factors faster and cheaper than conventional methods. These critical advances widen the use of biomechanical simulation tools in the research and potential personalized care of vascular diseases.

## Introduction

Hemodynamic factors, the stress and strain induced by pulsatile blood flow at the surface and body of a blood vessel, play a crucial role in vascular health and diseases mainly by altering the structure and functions of endothelial and smooth muscle cells. Among the hemodynamic factors, wall shear stress (WSS), the viscous shear applied to the endothelial cells due to blood flow, has been studied the most. Endothelial cells respond to WSS *via* a variety of mechanotransduction pathways (Davies, 1995; Chiu and Chien, 2011; Ando and Yamamoto, 2022; Tanaka et al., 2021). Cell-culture and animal studies have shown that different flow patterns trigger different endothelial responses. Generally, unidirectional flow is protective against atherosclerosis and neointimal formation, while complex, multidirectional flow is atherogenic and turns endothelial cells to become pro-inflammatory (Chiu and Chien, 2011). Complex, disturbed flow generates temporal and spatial gradients in luminal and wall hemodynamic parameters due to pulsatility in a cardiac cycle and the curvature, branches, and other geometric irregularities of the vasculature.

To understand the effects and mechanisms of hemodynamic factors on vascular structure and function, we first need to delineate the detailed *in vivo* three-dimensional (3D) blood flow characteristics and quantify the values of the hemodynamic forces. These data, however, cannot be obtained from analytical solutions of governing equations due to the irregular, complex geometry of the vasculature. Fortunately, with the rapid advancement of medical imaging and computational methods and power, computational fluid dynamics (CFD), finite element analysis (FEA), and fluid-structure interaction (FSI) simulations can be readily adopted and have been widely used to obtain a detailed flow field using vascular images obtained from computed tomography angiography (CTA), magnetic resonance imaging (MRI), ultrasound, intravascular ultrasound (IVUS), optical coherence tomography (OCT), and other imaging modalities. Values of the hemodynamic forces can then be derived using the detailed flow field.

**FIGURE 1:**
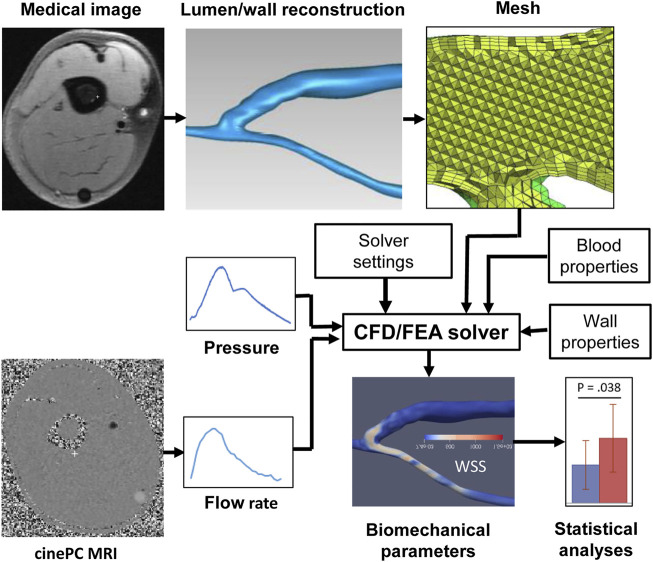
Pipeline of a typical image-based vascular CFD/FEA simulation. CFD, computational fluid dynamics, FEA, finite element analysis, PC MRI, phase-contrast magnetic resonance imaging, WSS, wall shear stress.

Biomechanical simulations using patient-specific anatomical and physiological data have been applied to study atherosclerosis in coronary (Abbasian et al., 2020; Guvenir Torun et al., 2021), carotid (Bennati et al., 2021), cerebral (Tanoue et al., 2011), and femoral (Wood et al., 2006) arteries; thoracic (Boccadifuoco et al., 2018a) and abdominal (Taylor et al., 1998) aortas; aortic aneurysms (Mariotti et al., 2021) and dissections (Cheng et al., 2014); cerebral aneurysms (Bazilevs et al., 2010); pulmonary arterial hypertension (Zambrano et al., 2018); bypass grafts (Sankaran et al., 2012), and arteriovenous fistulas for hemodialysis (He et al., 2013). In addition to research, CFD simulations have also been used clinically to derive the coronary fractional flow reserve values in stenotic coronary arteries from CTA images, avoiding invasive coronary angiography (Min et al., 2015). We will review the multiple steps that are generally followed in these simulations (Figure 1). Despite the great progress in the last 2 decades, some challenges still exist, and verification and validation must be performed to assess the simulation results (Tang et al., 2014). The emerging and exciting application of machine-learning techniques to biomechanics simulations will also be reviewed. Image-based biomechanical simulations have also been used to investigate the responses of vascular tissues to and predict the outcomes of the endovascular procedures, such as stenting of stenotic arteries and stent-grafting of aortic aneurysms and dissections (Auricchio et al., 2011; Hemmler et al., 2019; Raptis et al., 2019). Simulations of the interactions of these implants with blood vessels are further complicated by the implants and not reviewed here. Our current review can be complemented by other reviews of image-based computational cardiovascular biomechanics (Thondapu et al., 2017; Zhong et al., 2018; Liang et al., 2019; Cameron et al., 2020; Carpenter et al., 2020; Lipp et al., 2020; Lopes et al., 2020; Ong et al., 2020; Thiyagarajah et al., 2022).

## Pipeline of Image-Based Computational Biomechanical Simulations

### Imaging the Lumen

Imaging is performed to obtain the anatomical (lumen and wall) and physiological (flow) data of blood vessels that are needed for patient-specific biomechanical simulations. CTA and MRI have been the most popular modalities for vascular lumen imaging. During a spiral CTA scan, a narrow beam of x-rays is aimed at a patient and quickly rotates around the patient when the table on which a patient lies moves, producing signals that are collected by detectors opposite to the x-ray source and processed by a computer to generate cross-sectional images or slices of the body. These slices can then be digitally stacked together to form a 3D image of the patient. Multi-detector CTA uses iodinated contrast agents to enhance the vascular lumen for a quick, high-resolution scanning of the vasculature’s 3D geometry. For example, the spatial and temporal resolutions of the GE CT scanner can be 0.28 mm and 0.24 s, respectively. However, exposure to ionizing X-ray radiation, the risk of acute kidney injury from using contrast agents, and artifacts from nearby bone and metal implants are the main downside of CTA (Kim et al., 2010).

**FIGURE 2:**
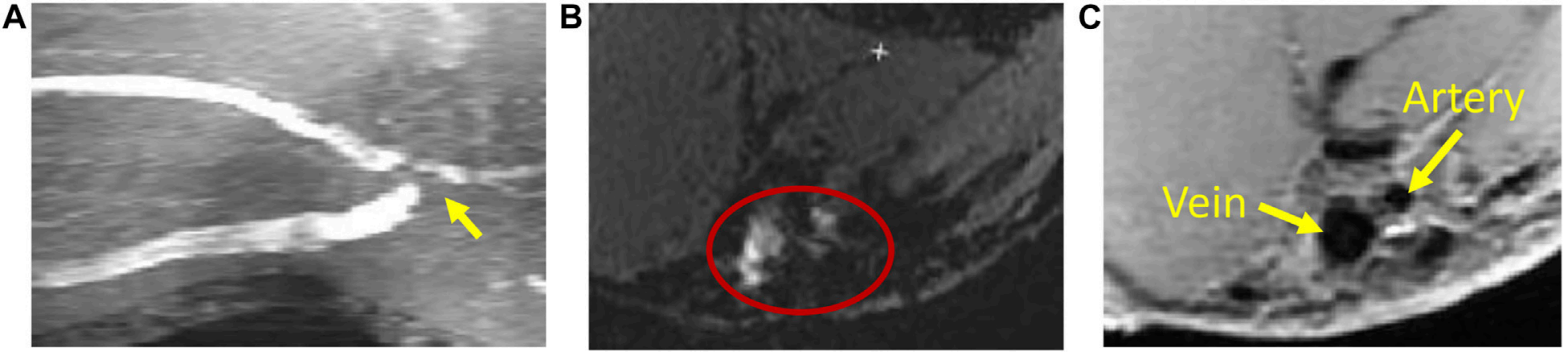
Magnetic resonance imaging of an arteriovenous fistula. **(A)** Maximal intensity projection of white-blood time-of-flight (TOF) images. At the anastomosis (the gray region pointed by a yellow arrow), the signal is void due to complex flow. **(B)** An example of the TOF slice close to the anastomosis (enclosed by the red ellipse) where the lumen is not clearly defined. **(C)** The black-blood image shows the lumen of the fistula vein and artery clearly (the black regions pointed by yellow arrows).

MRI uses a very strong magnetic field (typically, 1.5–3.0 T for clinical scanners) and radio frequency waves to create detailed images of the organs and tissues. The main magnetic field polarizes the magnetic spins of hydrogen nuclei. The radio frequency system excites the sample and detects the resulting MR signal, whose location is determined from the gradient coil system. The contrast between different tissues is determined by the rate at which excited hydrogen nuclei return to the equilibrium state. MRI allows for non-invasive tissue characterization because of its dependence on a variety of physical and chemical characteristics of the tissue, such as physical state, molecular motion, diffusion, chemical composition and concentration, and water content (Pooley, 2005). Through the technique of time-of-flight or double inversion recovery, the 3D lumen geometry can be obtained from MRI without using a contrast agent. For vasculature with complex blood flow, such as an arteriovenous fistula, the dark-blood images obtained by the double inversion recovery technique have a better quality than the white-blood images obtained by the time-of-flight technique, which is more susceptible to complex recirculating flows (Figure 2). Similar to CTA, MR angiography (MRA) can be enhanced using contrast agents, but without the risk of ionizing radiation.

### Imaging the Wall

The anatomical and compositional data of blood vessel walls and atherosclerotic plaques are also crucial components for studying vascular diseases and biomechanical simulations. Even though CT does not have an adequate contrast to differentiate vascular wall from adjacent perivascular tissues, it is commonly used for vascular calcification imaging because of the intrinsically higher signal intensity of calcium in CT. MRI is more versatile than CT. A variety of MRI techniques have been developed to non-invasively characterize vascular morphology and composition of atherosclerotic plaques, such as lipid core, fibrous cap, calcification, normal media, hemorrhage, and adventitia (Gold et al., 1993; Martin et al., 1995; Toussaint et al., 1996; Hatsukami et al., 2000). However, the spatial resolution of current clinical MRI scanners is limited (approximately 0.3 mm in-plane resolution with zero-filling interpolation at best) (He et al., 2013), and the thin fibrous cap (<65 μm), which is a crucial characteristic for the high-risk atherosclerotic plaque, cannot be clearly identified.

IVUS and OCT have been used to identify the components of the vascular wall at a higher spatial resolution, but they are invasive (Mantella et al., 2021). IVUS is a catheter-based procedure used to visualize the inside of a blood vessel in real time. Radiofrequency ultrasound waves, usually in the 30–60 MHz range**,** are emitted from the transducer at the catheter tip, and the return echo is also received by the transducer and conducted to an external computerized equipment to construct and display ultrasound images of a thin cross-sectional slice of the blood vessel. Virtual histology-IVUS, through spectral analysis of IVUS backscatter signals, can reveal the fibrous, fibro-fatty, necrotic-core, and dense-calcium regions of a plaque (Nair et al., 2002; Nair et al., 2007; Campos et al., 2015). However, even with a higher resolution (65–150 μm) than MRI, IVUS still cannot adequately measure the thickness of a thin fibrous cap.

Intravascular OCT has a higher resolution than IVUS. It utilizes back-scattered infrared light to generate high-speed and high-spatial-resolution (10–20 μm) images of blood vessels after using a contrast flush to clear the intraluminal blood (Shimamura et al., 2021). It can measure the thickness of a thin fibrous cap more accurately and identify the plaque composition (Brezinski et al., 1996; Roleder et al., 2015). However, OCT has a limited penetration depth through blood vessels, so the overall plaque burden cannot be measured. Taking the advantages of both IVUS (deep penetration) and OCT (high spatial resolution) by combining the two imaging modalities seems favorable and has been increasingly used (Li et al., 2014; Guo et al., 2018; Guo et al., 2021; Lv et al., 2021). The development of other hybrid intravascular imaging modalities, such as near infrared spectroscopy-intravascular ultrasound (NIRS-IVUS), further improves vascular wall imaging. NIRS can detect lipid composition by analyzing the near-infrared absorption properties of atherosclerotic plaques. A thorough discussion of hybrid intravascular imaging modalities can be found in Bourantas et al. (2017) and Kubo et al. (2022).

### Imaging the Flow

**FIGURE 3:**
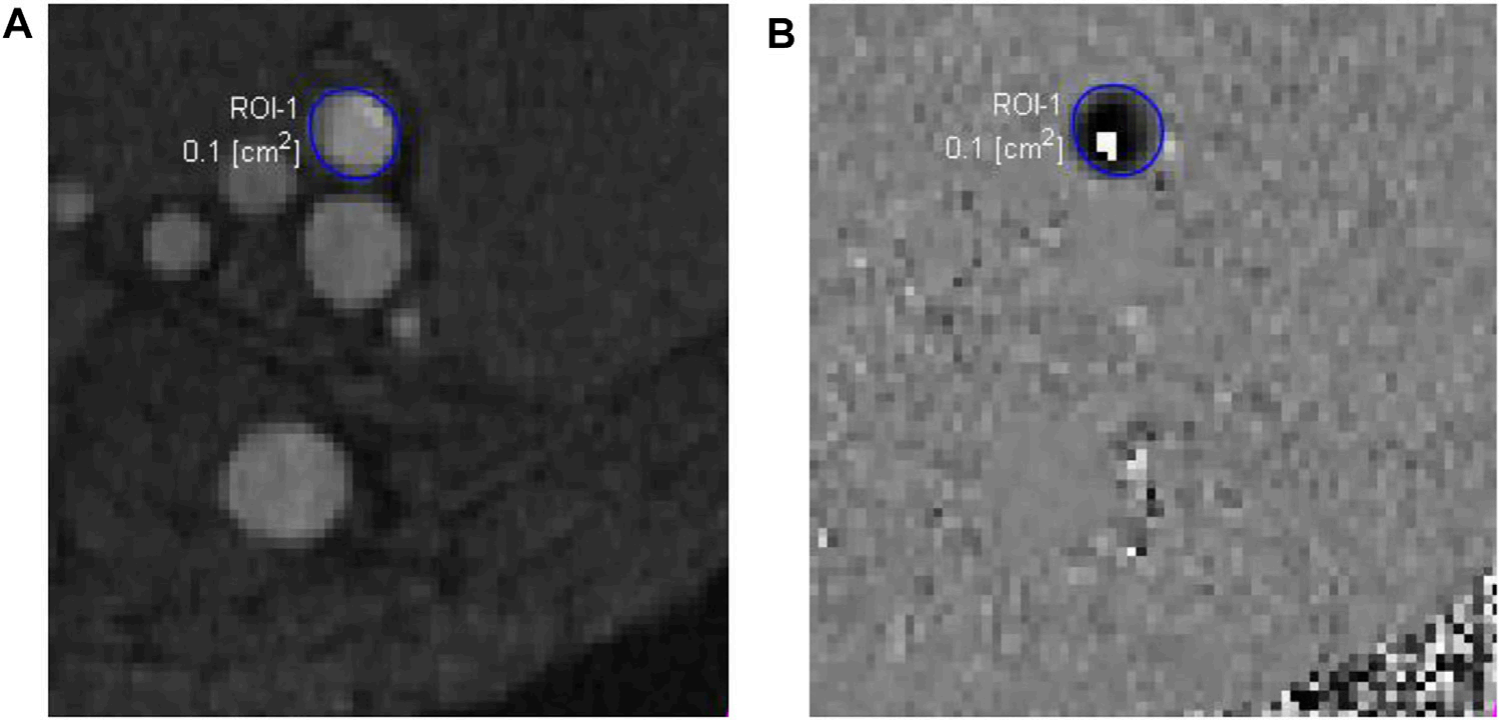
Phase-contrast magnetic resonance images of an arteriovenous fistula. The magnitude image **(A)** is used to draw a region of interest (ROI-1) for the proximal artery (enclosed by the blue circle), which may have very high flow velocity. In this case, the velocity encoding value, 250 cm/s, was not big enough, causing a phase-wrap artifact shown by the white pixels within the ROI-1 in the phase image **(B)**.

Compared to CTA, MRI also has the advantage of obtaining blood flow data using a phase-contrast sequence in either two dimensions (2D) or 3D. In 2D single-directional phase-contrast MRI, the through-plane velocity is measured by aligning the imaging plane perpendicular to the axis of the imaged blood vessel, assuming that the blood velocity is along the axial direction. The maximum intensity projection of time-of-flight images can be used to place the imaging plane. The velocity is encoded in the phase images using a velocity encoding value, which is the maximum velocity that can be measured without a phase-wrap artifact. When the phase-wrap artifact appears (Figure 3), the velocity encoding value needs to be increased. The flow rate can then be extracted from the phase and magnitude images using the free package Segment (Medviso AB, Lund, Sweden) (Bidhult et al., 2019) or other software. The magnitude images are used to define the lumen of imaged vessel. If the phase-wrap artifact is not severe, it can be corrected conveniently in Segment by unwrapping the affected pixels. When flow rates at several locations are needed, the scan must be repeated at each location, which is time consuming and burdensome to the patient and increases the possibility of patient-movement artifacts.

In the case of multi-directional flow measurement, four dimensional (4D, which is 3D in space plus time) phase-contrast MRI has been increasingly used to obtain time-resolved complex pulsatile blood flow velocities in three orthogonal directions within an acquired 3D volume (Markl et al., 2014; Azarine et al., 2019). Although a contrast agent is not required for phase-contrast MRI, the use of a contrast agent enhances the signal-to-noise ratio in the magnitude images and reduces noise in phase images, compared to scans without a contrast agent (Bock et al., 2010). Streamlines and velocity vectors can be used to visualize changes in blood flow pathways directly from the image data. In addition to visual flow analysis, one advantage of 4D flow MRI is that by post-processing the original data, flow rate through any plane across the volume can be obtained retrospectively. Therefore, when choosing the imaging plane orthogonal to the vessel axis is challenging, the number of measurement sites is large, and geometry of the vasculature is complex, 4D flow MRI is more accurate in flow rate quantification than 2D phase-contrast MRI by placing the measurement plane at the locations where helical or vortical flow does not exist or is mild.

In addition to MRI, duplex Doppler ultrasonography (DUS) is also commonly used to obtain blood flow data in peripheral vasculature. In DUS, an imaging sample volume (gate) is usually placed at the middle of the vessel to obtain the maximal velocity spectrum. In commercial ultrasound scanners, to derive the flow rate from the maximal DUS velocity spectrum, a parabolic velocity profile across the lumen is assumed, resulting in the mean velocity being half of the maximal velocity across the lumen based on the Poiseuille flow theory, which is only applicable to steady laminar flow in an infinitely long straight circular tube. However, arterial blood flow is pulsatile, and the velocity profile across the lumen depends on the Womersley number *α* ( $\alpha =R\sqrt{\rho \omega /\mu },\;$ where R is the lumen radius of the blood vessel, *ρ* is the blood density, *ω* is the angular frequency of blood flow pulsation, and µ is the blood dynamic viscosity) (Womersley, 1955). Consequently, a more accurate algorithm than that assuming a parabolic velocity profile has been developed by using the Womersley number to adjust the relationship between the mean and maximal velocities across the lumen (Ponzini et al., 2006). Of note, the Womersley velocity profile also depends on the assumption of a straight circular vessel. Therefore, an effort should be taken to choose a relatively straight circular segment of the blood vessel to measure flow rate. A more accurate lumen diameter measurement using a perpendicular cross-sectional view may also improve the flow rate measurement (He et al., 2018).

### Lumen and Wall Geometry Reconstruction

Image segmentation, i.e., extracting lumen and wall configuration from medical images, is one of the key steps for image-based computational biomechanics. The methods of segmentation depend on the imaging modality and image quality and can be manual, semi-automatic, or automatic. For images obtained by CTA and MRA using contrast agents, the image quality of the lumen is generally high, and implicit deformable models and level set methods can be used to extract the lumen quickly by specifying a few seed points in the images to define the region of interest (Antiga et al., 2008a). This family of methods uses spatial variation in image intensity, rather than absolute intensity, and is more robust than image intensity threshold-based methods. However, for time-of-flight and black-blood MRI, the image quality of the lumen also depends on the flow characteristics and is lower at the region with disturbed flow. In this case, manual delineation of blood vessels may be needed, even though this process is time consuming and the results are user dependent. Furthermore, the slice thickness of MR images may be ≥2 mm, which causes the reconstructed surface to become non-smooth at the region with a high curvature or large diameter changes along the length of the vessel. In the Amira software (Thermo Fisher Scientific, Waltham, MA), interpolation of segmented original slices to decrease the slice thickness may improve surface quality without increasing the workload of segmentation. Other software tools also may be considered, and users should find a tool that best suits their projects.

Because of the many options of MRI sequences and complex atherosclerotic plaque compositions, segmentation and image analysis of MRI data can be complicated and require special training (Kerwin et al., 2017). The vascular wall as a whole can be semi-automatically segmented from black-blood images with an adequate quality using deformable contour based on the initial inner and outer outlines manually drawn (Ladak et al., 2001). Different plaque components based on multi-contrast MRI can also be semi-automatically (Liu et al., 2012) or automatically (Adame et al., 2004) identified.

Virtual histology-IVUS offers automatic component identification and segmentation. Automatic or computer-aided plaque component identification, segmentation, and quantification have also been developed for OCT images (Athanasiou et al., 2014; Athanasiou et al., 2018; Guo et al., 2019; Lee et al., 2020). The impact of automated characterization of mixed plaque components in complex atherosclerotic lesions has also been evaluated recently (Olender et al., 2022). Reconstruction of a 3D vascular model from 2D IVUS and OCT slices need special consideration because of the 2D nature of these images. Reconstruction by fusing IVUS or OCT and biplane angiography or CTA images has been developed and used in clinical studies (Slager et al., 2000; Bourantas et al., 2005; van der Giessen et al., 2010; Samady et al., 2011; Wang et al., 2015b; Guo et al., 2018). Anatomical landmarks visible in both IVUS or OCT and angiography or CTA images are commonly used to estimate the orientation of the IVUS or OCT slices to facilitate the generation of the 3D model. A comprehensive review of the image data fusion methodologies has been published recently (Kilic et al., 2020).

### Meshing

The volumes of reconstructed lumen and wall need to be divided into small discrete elements, within which governing differential equations are solved. A high-quality mesh is a prerequisite for accurate simulation results. When creating a mesh, there are two main concerns, namely, the computational cost and the accuracy of simulation. A finer mesh gives more accurate results but requires more computational resources and time. Therefore, a common goal of meshing is to use less elements while achieving an acceptable accuracy. Due to the irregular and often complicated geometry of the vascular lumen reconstructed from medical images, an unstructured mesh is commonly used. When WSS is the target, which is often the case, a finer mesh at the blood-wall boundary, prismatic inflation layers, is required. To fill the rest of the lumen, hexahedral elements at the core can be created to reduce the number of elements if possible. Otherwise, tetrahedral elements in the core are generated. Since blood flow is more complex at the curved region and thus requires a finer mesh, a meshing algorithm using the local curvature to refine the mesh is advantageous if available. In Ansys Meshing, this is realized by adjusting the settings of the Curvature Size Function. Other software tools also may be considered, and users should find the details of the methods. In addition to more conventional tetrahedral and hexahedral mesh, polyhedral mesh has been demonstrated to be better than tetrahedral mesh in reducing the number of elements, reaching convergence faster, and achieving WSS patterns that have less artifacts and thus are more homogeneous (Spiegel et al., 2011). To ensure an adequate mesh is created, a mesh-independence analysis should be performed based on the most important parameters in question, which are WSS parameters in most cases. The grid convergence index is recommended for the uniform reporting of grid refinement studies (Roache, 1994).

Meshing of the wall with advanced atherosclerotic plaques may be challenging due to the complex and irregular geometries of the different components. A component-fitting technique has been developed to generate mesh that can overcome this challenge (Yang et al., 2009). Using this technique, the 3D plaque components are divided into multiple small volumes to cover the irregular plaque geometry; a mesh for each volume is then generated. This approach is, however, labor intensive. More sophisticated and automatic mesh generation techniques have also been developed using an octree-based isocontouring method (Zhang et al., 2010).

### Mechanical Properties

CFD/FSI simulations of blood flow require blood viscosity. The viscosity of whole blood varies with the hematocrit, leukocyte and platelet counts, plasma protein composition and concentration, as well as the shear rate. At least 15 non-Newtonian blood rheological models have been proposed to take into account the shear-thinning property (Abbasian et al., 2020). These models have different popularity; the Casson, Carreau, Carreau-Yasuda, power-law, and Quemada models are mostly used. When the shear rate is above 100 s^−1^, blood behaves similarly to a Newtonian flood.

FSI simulations of blood flow require mechanical properties of blood vessel wall. Since blood vessels have complex, nearly incompressible, non-homogeneous, anisotropic, non-linear, and viscoelastic (creep, stress relaxation, and hysteresis) material behaviors, it is very difficult, if not impossible, to obtain the *in vivo* material parameters describing these behaviors. *In vivo*, *ex vivo*, *in vitro*, and *in silico* methods have been used to characterize these behaviors and derive purely phenomenological or structure-motivated constitutive models that hold only under specific conditions of interest (Holzapfel and Ogden, 2010).

Pulse wave velocity has been measured by tonometry, Doppler, or oscillometry to quantify arterial stiffness *in vivo* by many studies because its measurement is minimally invasive and inexpensive, but it only offers a single average value for the artery segment between the measuring sites, neglecting any regional variation and perivascular tethering effects (Hodis and Zamir, 2011). Medical imaging techniques have been used to obtain vascular mechanical properties. But since the *in vivo* clinical images only represent the physiologically loaded states, inverse FEA approaches, which could be used to obtain the unloaded configuration, are needed for *in vivo* material parameter identification. Therefore, medical images obtained from various imaging modalities have been combined with inverse FEA to derive *in vivo* vascular mechanical properties. For example, the *in vivo* aortic elastic properties of ascending thoracic aortic aneurysm have been identified from gated CT scans using an inverse approach (Liu et al., 2019a). An iterative procedure has also been developed to identify coronary artery mechanical properties by matching both maximum and minimum *in vivo* Cine IVUS lumen circumferences (Guo et al., 2017; Wang et al., 2021). Also using inverse FEA, the mechanical properties of infrarenal abdominal aorta and its peri-aortic structure have been assessed using displacement encoding with stimulated echoes (DENSE) MRI (Bracamonte et al., 2020). However, some assumptions were made when using inverse FEA. For example, the diastolic configuration was treated as the zero-strain reference, and the aortic wall was commonly assumed to be homogeneous (Bracamonte et al., 2020). Inverse FEA methods with more realistic conditions are needed. Recently, to overcome the previous limitation of homogenized or simplified material representations, an inverse FEA approach was developed to derive non-linear material properties of heterogeneous coronary plaque components using OCT imaging data acquired at differing pressures by incorporating interfaces between various intra-plaque components into the objective function (Narayanan et al., 2021). The importance of including multi-material plaque components has also been demonstrated by the greatly varied lesion mechanical responses (Kadry et al., 2021).

Due to their large size and a propensity for aneurysm formation and wall dissection, aortic tissues have been most extensively studied *ex vivo* (Di Martino et al., 2006; Vande Geest et al., 2006; Cebull et al., 2020; Jadidi et al., 2020). Uniaxial and biaxial tensile tests of explanted tissue strips from patients demonstrate that aortic tissues are stronger and stiffer in the circumferential than axial direction (Sokolis et al., 2012; Pichamuthu et al., 2013; Jadidi et al., 2021a). This behavior is due to preferential alignment of collagen fibers in the circumferential direction. Since a single elastic modulus value is inadequate to describe the non-linear behavior in the whole range of deformation, various forms of strain energy density functions based on deformation invariants are used to describe arterial hyperelastic behaviors under large deformation. Exponential and polynomial strain energy density functions are the most popular. Some constitutive models have also been developed to reflect the microstructural data, especially the directions of collagen fibers obtained from various optical microscopic imaging techniques (Holzapfel et al., 2000; Gasser et al., 2012; Jadidi et al., 2021b).

### Boundary Conditions

Appropriate boundary conditions define the effect of the truncated vasculature and perivascular tissues within the simulated domain and are the key to obtaining accurate simulation results (Campbell et al., 2012; Gallo et al., 2012; Xu et al., 2018). Boundary conditions are needed at all the inlets and outlets. Ideally, patient-specific simulations require known velocity and blood pressure profiles at all nodes of the inlets, outlets, and wall. The velocity and flow rate can be acquired by MRI and ultrasound non-invasively. Accurate blood pressure measurement at specific locations, however, requires intravascular access by a pressure transducer and is usually unavailable. Except 4D flow MRI measurement, the 3D velocity profiles at the inlets are unknown using other modalities. Therefore, it is common to apply the average velocity (plug flow) obtained by dividing the flow rate with the cross-sectional area at the added, extended straight inlet to achieve a fully developed flow at the location of the original inlet, but this approach may be problematic as actual flow may be skewed at the inlets that have a significant curvature and geometric irregularity. When 4D MRI velocity data are available, it is better to specify the velocity component at each direction at the inlet directly than to assume idealized velocity profiles derived from the measured flow rate (Morbiducci et al., 2013).

When patient-specific data are not available, several strategies have been devised to achieve reasonable results. Setting a zero pressure at multiple outlets has been used, but it is not a good practice because it may fail to reproduce physiologically relevant flow and pressure features (Pirola et al., 2017; Chnafa et al., 2018). Using typical or population-averaged flow rates and waveforms is common, but still not ideal because of the heterogeneity among patients. Alternatively, since lumen area or diameter data are more readily available from non-invasive clinical images, the inlet flow rate and flow split through branches can be estimated using lumen area or diameter data and various scaling laws, such as Murray’s law based on minimum energy theory (Murray, 1926), or developed from measured data (Cebral et al., 2008; van der Giessen et al., 2011; Tricarico et al., 2020). In CFD simulations assuming a rigid wall, the velocity boundary condition at the wall is generally set as no-slip or zero-velocity.

In FSI simulations, blood pressure is important for accurate wall stress and strain results. When blood pressure is not available, lumped-parameter Windkessel models representing the impedance of truncated downstream vasculature can provide reasonable pressure values when the flow and parameters of the Windkessel models are appropriate (Westerhof et al., 2009; Pirola et al., 2017). However, patient-specific Windkessel parameters require patient-specific pressure and flow waveforms, which are not available in this case. Assumptions must be made to use Windkessel parameters obtained from other sources. As a way of considering the compliance, resistance, and especially wave reflection of the downstream truncated vasculature, an elastic tube terminated with a rigid contraction has been added to the outlet as a part of the computational domain (Pahlevan et al., 2011).

For structural analysis, the constraints at the nodes of the inlets and outlets differ among studies. In some studies, the translational motion was fixed while rotational motion was unconstrained for the nodes at the inlet and outlets (Nathan et al., 2011; Gomez et al., 2021), but the end nodes did not have any moving freedom (Pasta et al., 2013). In another study, nodes at the proximal end were allowed to deform only in the radial direction, while the distal ends were fixed in all directions (Martin et al., 2015).

Blood vessels are constrained radially by the surrounding perivascular tissues, and this constraint reduces the distensibility and intramural stress of an artery (Vonavkova and Horny, 2020), but it is a challenge to prescribe the *in vivo* perivascular boundary conditions. Although many studies simply ignore the perivascular constraint, some studies have attempted to model it. In one study, the radial constraint was quantified as an effective perivascular pressure applied to the outer surface of adventitia, which could be ≥50% of the intravascular pressure (Liu et al., 2007). In another study, the effect of the perivascular tissue was applied as an effective pressure waveform at the external wall of carotid arteries (Soleimani et al., 2021) or modeled as a heterogeneous elastic foundation boundary condition, which was implemented as a collection of unidimensional springs attached to the adventitial surface (Bracamonte et al., 2020).

### Solution Strategies

Depending on the study objectives, a few software packages can be chosen to solve the governing differential equations of blood flow and vessel wall deformation. The common packages use finite volume (Ansys Fluent and CFX, Siemens STAR CCM+, OpenFoam) or finite element (COMSOL, SimVascular, Crimson) methods for CFD; FEA (Ansys Mechanical, Simulia Abacus, SimVascular) for structural wall stress analysis; Arbitrary Lagrangian-Eulerian (ALE) formulation (Adina, SimVascular, Simulia Abacus) or coupling the CFD and FEA solvers (Ansys Workbench) for FSI. If the focus is on hemodynamics, not detailed structural stress and strain distributions, the coupled momentum method is an efficient alternative to ALE formulation (Figueroa et al., 2006). In this method, wall deformation is assumed to be small; therefore, the fluid mesh is not updated. The vessel wall is based on a membrane model. This simplified method yields valid results in cases where the assumptions of small deformation and thin walls are indeed valid.

Several choices need to be made to balance the computational effort and accuracy. High-order numerical schemes and appropriate time-step size and residual errors should be used in addition to an appropriate mesh size (Khan et al., 2015; Dennis et al., 2017). Laminar flow is commonly assumed for most blood flow under physiological conditions, but transition to turbulence may occur in normal aorta (Ha et al., 2018), aorta with aortic valve stenosis (Manchester et al., 2021), stenotic arteries (George et al., 2008; Lantz et al., 2013; Andersson et al., 2017), intracranial aneurysms (Valen-Sendstad et al., 2011), and arteriovenous grafts (Lee et al., 2007) or fistulas (Stella et al., 2019). It has been demonstrated that flow instability can only be revealed under high spatial and time resolutions (Baek et al., 2010; Valen-Sendstad and Steinman, 2014). In the studies of turbulent blood flow, traditional Reynolds-averaged Navier–Stokes (RANS) equations-based turbulence models (k–ɛ and k–ω) (George et al., 2008; Perinajová et al., 2021), large eddy simulation (LES) (Lantz et al., 2013; Andersson et al., 2017; Stella et al., 2019; Manchester et al., 2021) and direct numerical simulation (DNS) (Lee et al., 2007; Valen-Sendstad et al., 2011; Arzani et al., 2012) have been used. LES directly resolves large-scale, at the size of mesh grid, velocity fluctuations. It incorporates the dissipative energy loss induced by turbulent eddies at the sub-grid level and can therefore model laminar, transitional, and turbulent features, which may all be exhibited in the different phases of the pulsatile blood flow during a cardiac cycle. Because DNS directly solves Navier–Stokes equations numerically for the scales of all turbulent eddies without using any turbulence model, it requires much more number of mesh elements and computational resources and time than LES and is thus seldom used for complex blood flow simulations.

Image-based FEA wall stress analysis of blood vessels needs special treatment of the vascular geometry because the geometry obtained from medical images has a deformed configuration by intraluminal pressure and axial stretch. Blood vessels are stretched circumferentially, radially, and axially at the *in vivo* loaded state. In addition, there are residual stresses and strains even under the unloaded state (Chuong and Fung, 1986). However, the ideal reference configuration for FEA requires zero stresses and strains everywhere within blood vessels. Therefore, the *in vivo* vessel geometry needs to be shrunk circumferentially or radially and axially to obtain its unloaded state, which then needs to be numerically cut open radially to release the residual stresses and strains to obtain the ideal reference configuration. The *ex vivo* images of carotid arteries at a unloaded state have been stretched axially and circumferentially to match the *in vivo* MRI images in FSI simulations (Huang et al., 2009). However, the *ex vivo* unloaded state is unavailable in most patient-specific simulations. Therefore, various methods have been developed to drive the unloaded or stress-free configuration from the geometry obtained from *in vivo* clinical images. Assuming known material properties, these methods either 1) estimate the pre-stress field on the *in vivo* configuration, then depressurize the FE model to obtain the unloaded geometry (Gee et al., 2010; Weisbecker et al., 2014; Maas et al., 2016); 2) estimate the unloaded configuration by adjusting an initial geometry and running forward FE simulations (Raghavan et al., 2006; Bols et al., 2013; Riveros et al., 2013); or 3) use an inverse FE formulation (Lu et al., 2007). These methods require many FE iterations to converge and are therefore time consuming.

### Biomechanical Parameter Extraction and Statistical Analysis

To quantify WSS’s magnitude, multi-directionality, and pulsatility, the parameters that are most commonly extracted to characterize the local flow conditions experienced by endothelial cells at the vessel wall include time-averaged WSS magnitude over a cardiac cycle (TAWSS), maximum WSS within a cardiac cycle (WSSmax), oscillatory shear index (OSI) (He and Ku, 1996), relative residence time (RRT) (Himburg et al., 2004), WSS spatial and temporal gradients, axial WSS (WSSax), the secondary component of WSS (WSSsc), ratio of WSSsc to WSSax, transverse WSS (TAWSStrans), and cross-flow index (CFI) (Colombo et al., 2021). WSSax is the WSS component aligned with the tangent to the vessel centerline, while WSSsc is the other component of WSS in addition to WSSax (Morbiducci et al., 2015). TAWSStrans is the average over the cardiac cycle of WSS components perpendicular to the temporal mean WSS vector (Peiffer et al., 2013b). CFI is the normalized TAWSStrans by the WSS magnitude (Mohamied et al., 2017). Note that depending on the geometry, the temporal mean WSS vector at a location does not necessarily align with the direction tangent to the vessel centerline.

A recent promising advance is the development and application of an Eulerian method for obtaining WSS vector field topological skeleton that has a strong link with features of disturbed flow, such as flow separation, stagnation, impingement, and reversal (Mazzi et al., 2020; Mazzi et al., 2021). Based on the dynamical system theory, the WSS topological skeleton consists of fixed points where the WSS value is zero and manifolds that link the fixed points. In the blood flow field, the stable or unstable manifolds identify regions where the WSS vector exerts an expansion or contraction action on the endothelial cells that are potentially important for developing vascular diseases. The cycle-averaged WSS topological skeleton also relates to the fluid-phase mass transport of solutes near the wall (Arzani and Shadden, 2018). Clinically, high temporal variation of WSS contraction or expansion and high fixed-point residence times weighted by WSS contraction or expansion strength at 1 month after endarterectomy have been found to predict long-term carotid bifurcation intima-media thickness, independently from the exposure to low WSS (Morbiducci et al., 2020). The associations of WSS topological skeleton features with vascular pathophysiology need further studies.

The common biomechanical parameters of the vessel wall extracted from FEA are maximum principal stress for studying the sites of plaque rupture (Tang et al., 2009; Costopoulos et al., 2019) and aortic aneurysms (Martin et al., 2015), peak longitudinal and circumferential wall stresses for aortic aneurysms (Gomez et al., 2021) as well as Von Mises stress in normal aortas (Nathan et al., 2011) and aortic aneurysms (Rissland et al., 2009). Using FEA-derived peak stress, a multifactorial stress equation of peak stress that is based on the analysis of plaque morphological parameters has also been developed recently (Hartman et al., 2021). These plaque morphological parameters include fibrous cap thickness, necrotic core angle, necrotic core thickness, lumen area, and necrotic core calcium and plaque areas. This methodology has the potential of obtaining the peak stress within a plaque rapidly because the detailed FEA is not required.

The high spatial resolution of the data obtained from CFD or FEA simulations bring challenges in statistical analysis that investigates the relation between the focal nature of vascular diseases and biomechanical factors because these data are spatially autocorrelated (i.e., the data are more like each other when they are closer in space). Treating all the data points as independent violates the assumption of standard statistical tests and will artificially augment the sample size and obtain extremely small *p* values even for a very small effect size. Several methods with different mathematical complexities have been proposed to consider the spatial correlation (Peiffer et al., 2013a; Rowland et al., 2015). These methods are decorrelation length-based sampling, Dutilleul’s modified *t*-test, iterative amplitude adjusted Fourier transform, dual-tree complex wavelet transform, and a bootstrap approach.

Linear mixed-effects regression models have also been used to accommodate the clustering of the multiple slices within a blood vessel. A mixed-effects regression model incorporates both fixed and random effects. The random effect considers correlations among the data points due to clustering within a vessel (He et al., 2020; Hoogendoorn et al., 2020), within a plaque (Costopoulos et al., 2019) or within the slices (Wang et al., 2015a). The spatial covariance structure can also be specified in a mixed-effects model. In a study of vein bypass graft remodeling, a one-dimensional exponential spatial covariance structure was used for the strong autocorrelation among lumen diameters along the sections in each graft (He et al., 2020). Another concern is the correlation between data obtained at multiple time points from the same patient in a longitudinal study. Linear mixed-effects models can also be used in this case with the patient as a random factor (Colombo et al., 2021).

Human studies have been performed to assess the predictive power of different biomechanical parameters on the initiation, development, and rupture of atherosclerotic plaques. Low and oscillatory WSS has been widely recognized to be the key hemodynamic factor in the initiation and development of atherosclerosis. TAWSS had a higher sensitivity of predicting plaque location in both right and left coronary arteries than average WSS gradient, OSI, and RRT (Knight et al., 2010; Rikhtegar et al., 2012). However, a systematic review found that the evidence for the low/oscillatory shear theory is less robust than commonly assumed (Peiffer et al., 2013c). The definition of low WSS is important and may affect the conclusion (Hartman et al., 2021).

## Verification, Validation, and Uncertainty Quantification

All image-based simulations involve uncertainties and potential errors related to every input needed in the modeling pipeline, including geometry, boundary conditions, mechanical properties, unavailable input parameters that are difficult or unethical to obtain from study subjects, solver settings, and necessary modeling assumptions (Steinman and Migliavacca, 2018; Valen-Sendstad et al., 2018; Steinman and Pereira, 2019). The sizes of these variations and their impact on predicted biomechanical factors need to be assessed through VVUQ. In the context of vascular biomechanical simulations, verification is performed to assess if the numerical simulations solve the simplified mathematical description of the vascular system correctly, and validation is implemented to determine if the model accurately represents the *in vivo* conditions. Uncertainty quantification evaluates how variations in the physical and numerical parameters affect biomechanical factors obtained from simulations. Many VVUQ studies in vascular biomechanical simulations have been performed (Steinman and Migliavacca, 2018). However, since the *in vivo* true values are usually unknown, most of these studies can only evaluate the relative differences compared to the results obtained from other methods.

### Geometry (Imaging and Segmentation)

The imaging uncertainties depend on the imaging hardware, image acquisition protocols, techniques of image reconstruction from raw data, and specific characteristics of the patient. The composite effects of obliqueness, in-plane resolution, and voxel anisotropy on the accuracy of black-blood MRI-derived wall thickness measurements at the carotid bulb have been studied (Antiga et al., 2008b). Thick-slice axial acquisitions can result in artificial wall thickening due to its obliqueness to the imaging plane. Reduction of in-plane resolution can also exaggerate wall thicknesses by up to 50%.

The variation of lumen segmentation algorithms of five intracranial aneurysms from 3D digital subtraction angiography (DSA) images among the 26 participating groups has been assessed (Berg et al., 2018). Although qualitative similarity of the aneurysm representations was obtained, the inter-group differences of the aneurysm volumes, ostium surface areas, and morphology parameters (undulation and non-sphericity) were up to 20%, 30%, and 25%, respectively. These morphological variations led to 28%–51% variation in TAWSS, which may lead to an inappropriate interpretation of the simulation results (Voß et al., 2019). In another study using high-resolution OCT-derived geometry as the ground truth, the segmentation of coronary CTA images showed that the measurement uncertainty in minimum lumen diameter had the largest impact on CFD simulation-derived fractional flow reserve (FFR) (Sankaran et al., 2016). The CFD simulations based on coronary CTA images were found to overestimate the absolute TAWSS values than those based on IVUS/OCT images, although the WSS patterns were similar and the correlation and concordance were high (Eslami et al., 2021). Polynomial chaos expansion is another method for a global sensitivity analysis. It uses a stochastic approach to obtain continuous response surfaces of the hemodynamic parameters starting from a few deterministic simulations and is computationally more efficient than a Monte Carlo approach. Furthermore, using the method of polynomial chaos expansion for a global sensitivity analysis, it has been found that the sensitivity to geometry may be different during different instants of the heartbeat and in different vascular regions (Xu et al., 2021).

### Blood Properties

Patient-specific blood properties are almost never available for biomechanical simulations, even though blood viscosity can vary up to 20% among individuals (Box et al., 2005). The commonly used viscosity values for the Newtonian model may vary by 10% among different patient groups (Valen-Sendstad et al., 2018). The effects of different blood rheological models on hemodynamics have been evaluated in stenotic carotid (Mendieta et al., 2020) and coronary (Abbasian et al., 2020) arteries and intracranial aneurysms (Oliveira et al., 2021). It has been found that the effects of different blood rheology models on simulation results depend on the specific hemodynamic parameters, and the difference in hemodynamic parameters can be more than 50% compared to the Newtonian model. Using a non-Newtonian blood viscosity model in a simulation requires to update viscosity after each iteration, so it takes longer to finish simulations, and it is common to use a constant blood viscosity value if the blood flow velocity and shear rate are expected to be high. Using a Newtonian model also is more appropriate when the overall flow pattern or the mean WSS parameters are investigated; the non-Newtonian model is necessary when the low WSS region is the focus, especially for arteries with severe stenosis. When there is stenosis, the region with high velocity and WSS will not be affected significantly by the use of non-Newtonian models.

### Wall Properties

In FEA, it is common to assume a constant wall thickness along the length of a blood vessel, but this assumption may be problematic. For example, this led to a significantly different wall stress distribution from that of a patient-specific model using micro-CT-measured wall thickness of an intracranial aneurysm, although the average wall stresses may be similar (Voß et al., 2016). Therefore, use of patient-specific regionally varying wall thickness is recommended for estimating peak biomechanical parameters, especially when the wall thickness is expected to be non-uniform, such as with atherosclerosis, aneurysm, and stenosis (Raut et al., 2013). Additionally, using an anisotropic material model may estimate much higher wall stresses compared to the isotropic and uniform-thickness mode (Mesri et al., 2017). Using a more general non-symmetric collagen fiber dispersion model in arterial walls (Holzapfel et al., 2015) or considering intima heterogeneity (Akyildiz et al., 2018) is needed for better describing the arterial mechanical behavior. As such, the above studies demonstrate the importance of using more realistic wall properties.

In CFD simulations, a rigid wall is most commonly assumed, but the variability in vessel lumen diameter during a cardiac cycle is approximately 5%–10% in most major normal arteries (Taylor et al., 1998). If not specifically cardiac-gated at systole, the images are more likely to be taken at diastole because systole is shorter than diastole. Therefore, the lumen size in simulations using a compliant wall is larger than that using a rigid wall, resulting in smaller velocity and WSS in simulations using a compliant wall than those using a rigid wall, although the distribution patterns of WSS parameters are similar (Kim et al., 2008). On average, the TAWSS of the compliant-wall simulation has been found to be 21.5% lower than that of a rigid-wall simulation for a hemodialysis arteriovenous fistula (McGah et al., 2014). Other studies reported a similar magnitude of difference (to be 25%) in WSS in an idealized carotid bifurcation model (Perktold and Rappitsch, 1995) or smaller (13%) in aortas reconstructed from MR images (Stokes et al., 2021). Furthermore, the effects of assuming a rigid wall on hemodynamic factors may not be uniform across different regions. At the anastomosis of an arteriovenous fistula with a more disturbed flow, the WSS difference between the compliant and rigid walls can be up to 58% (McGah et al., 2014). As expected, decreasing the Young’s modulus of the aortic wall causes a more significant underestimation of the peak flow rate (Boccadifuoco et al., 2018a). Nevertheless, previous data suggest that the effect of using a rigid wall in image-based simulations may be less than that due to uncertainties in geometry and boundary conditions (Lee and Steinman, 2007). The validity of the qualitative and quantitative relations between WSS parameters and vascular diseases obtained from a rigid-wall simulations needs to be assessed.

### Boundary Conditions

Using a statistical model of blood flow in internal carotid artery in CFD simulations, it has been observed that flow waveform variations at the inlet of internal carotid artery have a limited influence on the TAWSS on the saccular intracranial aneurysm surface, but the internal carotid artery flow waveform strongly affects WSS directionality in regions where the flow is highly multidirectional (Sarrami-Foroushani et al., 2016). The impact of uncertainties in the values of Windkessel model parameters at the outlets on the simulation results of an ascending thoracic aortic aneurysm has been quantified using generalized polynomial chaos expansion (Boccadifuoco et al., 2018b). Again, the results show that the uncertainties in the selected outflow parameters have only a moderate effect on TAWSS but may lead to significant variability of the instantaneous WSS in regions with complex flow. Using a similar method, it has been found that the uncertainty of the Windkessel resistance parameters at the outlets of a thoracic aorta with a coarctation induces a remarkable variability on the flow rate waveform at the peak systole but has a slighter effect on the pressure gradient across the coarctation (Antonuccio et al., 2021).

### Validation of Computational Fluid Dynamics Simulations

Some *in vitro* phantom-based experiments and *in vivo* measurements by MRI have been performed to validate the results obtained from CFD simulations. Using a compliant silicone phantom aneurysm model and 3D rotational angiogram, the reliability of the CFD simulation was confirmed by comparing the actual and virtual angiograms obtained from CFD simulations of the contrast concentration (Sun et al., 2010). Another study using a rigid, patient-specific phantom of a complex abdominal aortic aneurysm showed a high degree of agreement between numerically simulated and experimentally measured velocity fields at selected slices by MRI (Kung et al., 2011). Furthermore, the pressure waveforms also had an excellent agreement with only a 3.8% difference between measured and predicted root-mean-square pressures at the light exercise condition (Kung et al., 2011). An *in vivo* 4D-flow MRI study showed the necessity of using turbulent models in simulating thoracic aortic flow (Miyazaki et al., 2017). Also based on 4D-flow MRI, it has been demonstrated that a compliant-wall computational model is needed to show the time lag at the outlets of thoracic aorta with respect to the inlet flow waveform found in MRI data (Boccadifuoco et al., 2018a). However, MRI data may not be ideal for validating CFD results. For example, the WSS and energy loss calculated from MRI data were less than those obtained from CFD simulations around the aortic arch due to the limitation of MRI spatial resolution (Boccadifuoco et al., 2018a).

## Machine Learning

Promising artificial intelligence (AI) and machine learning (ML) methods have been increasingly used in various aspects of vascular biomechanics research. These methods include imaging (Henglin et al., 2017; Rutkowski et al., 2021); segmentation of images obtained from different imaging modalities (Nasr-Esfahani et al., 2018; Guo et al., 2019; Livne et al., 2019; Zhao et al., 2019; Bajaj et al., 2021; Comelli et al., 2021; Tian et al., 2021); estimation of constitutive parameters *in vivo* (Liu et al., 2019b) or for harvested vascular tissues (González et al., 2020; Liu et al., 2020); estimation of the zero-pressure geometry of human thoracic aorta from two pressurized geometries of the same aorta at two different blood pressure levels (Liang et al., 2018); prediction of hemodynamics in human thoracic aorta trained on CFD data (Liang et al., 2020) or stresses within atherosclerotic walls trained on FEA data (Madani et al., 2019); computation of a probabilistic and anisotropic failure metric of the aortic wall (Liu et al., 2021); and prediction of plaque vulnerability (Cilla et al., 2012; Guo et al., 2021).

Deep learning in medical image analysis is a branch of ML mainly based on convolutional neural network (CNN) methodology (Litjens et al., 2017). When a neural network contains multiple layers between the input and output, it is considered a deep neural network (DNN). Vascular segmentation using well-validated deep learning methods can automatically extract the vascular structure quickly and without operator bias. To develop the deep learning model using a supervised learning approach, a large training dataset that is usually segmented manually or semi-automatically by experts is required. The most popular deep CNN used in medical image segmentation is U-Net (Ronneberger et al., 2015). U-Net has accurately segmented the images of ascending thoracic aortic aneurysm (Comelli et al., 2021) and arteries in the brain (Livne et al., 2019). Other tools, E-Net and V-Net, have also been applied successfully to ascending thoracic aortic aneurysm and coronary artery, respectively (Comelli et al., 2021; Tian et al., 2021).

Conventional image-based CFD, FEA, and FSI simulations are time consuming, limiting their potential clinical applications. Training ML algorithms using CFD and FEA simulations to combine the two methods can help generate results much faster without considerably affecting the performance. As an example, a DNN model could predict the steady velocity magnitude and pressure fields in a thoracic aorta with an average error of 2.0 and 1.4%, respectively, in one second (Liang et al., 2020) or calculate the FFR values with an excellent correlation to CFD predictions in a few seconds (Itu et al., 2016; Coenen et al., 2018). Also using DNN, the predicted peak von Mises stress magnitude in atherosclerotic artery has had an average error less than 10% (Madani et al., 2019). However, the great variations in geometry and boundary conditions among patients make data-driven models difficult to be trained in high-dimensional feature spaces. Further development of fast and real-time CFD and FEA simulations accelerated by ML algorithms may help realize clinical application potential of these biomechanical tools (Phellan et al., 2021).

## Conclusion

Image-based simulation of the vasculature biomechanics is an active research area. From the perspective of biomechanics and mechanotransduction, it aims to partially reveal the mechanisms of the heterogeneity in the initiation, progression, and treatment response of vascular diseases in different patients. However, a completely personalized simulation, including specific, high-fidelity lumen and wall geometry, flow and pressure boundary conditions at the inlets and outlets, blood and wall properties, and interaction with perivascular tissues, is very challenging and not practical even with recent great advances in imaging and computational algorithms and power. The use of simplified models is necessary, but the validity of these simplified models needs to be thoroughly evaluated. The development and application of novel ML algorithms to all aspects of the CFD, FEA, and FSI pipelines have the potential to accelerate the application of the biomechanical analysis tools to the research and perhaps the treatment of vascular diseases. The future clinical applications may include prediction of the sites with future cardiovascular events, such as formation or rupture of atherosclerotic plaques or aneurysms, and thrombosis formation. In combination with technical advancements, large, prospective, image-based clinical studies are needed to evaluate the capability of biomechanical parameters in predicting hard-defined clinical endpoints (Gijsen et al., 2019).
